# Effects of TiO_2_ nanoparticles on nutrition metabolism in silkworm fat body

**DOI:** 10.1242/bio.015610

**Published:** 2016-05-16

**Authors:** J. H. Tian, J. S. Hu, F. C. Li, M. Ni, Y. Y. Li, B. B. Wang, K. Z. Xu, W. D. Shen, B. Li

**Affiliations:** 1 School of Basic Medicine and Biological Sciences, Soochow University, Suzhou, Jiangsu 215123, China; 2 National Engineering Laboratory for Modern Silk, Soochow University, Suzhou, Jiangsu 215123, China

**Keywords:** Silkworm, Fat body, TiO_2_ NPs, Nutrient metabolism

## Abstract

Silkworm (*Bombyx mori*) is an important economic insect with a fat body that plays a crucial role in the storage and transfer of nutrients. It is also known that TiO_2_ nanoparticles (NPs) can improve feed efficiency and promote silk protein synthesis in the silkworm. In this study, we profiled gene expression in the silkworm fat body after TiO_2_ NP treatment, validated the major RNA-seq findings, and determined the contents of trehalose and triglyceride, the activity of lipase, and the amount of total proteins. RNA-seq analysis revealed that TiO_2_ NP treatment caused significant expression changes in 341 genes (*P*≤0.01), 138 of which were upregulated while the other 203 were downregulated. The expression levels of two target genes in the insulin signaling pathway and two protein metabolism-related target genes, three lipid metabolism-associated target genes, two carbohydrate metabolism related target genes and expression levels of seven heat shock protein genes were increased, and that of threonine dehydratase gene and fatty acid transport protein gene were decreased. The RNA-seq results of 16 genes were validated by quantitative real-time PCR. The lipase activity, content of trehalose, and amount of total proteins were elevated by 3.86-fold, 1.34-fold, and 1.21-fold, respectively, and the content of triglyceride was decreased by 0.94-fold after TiO_2_ NP treatment. These results indicated that TiO_2_ NPs activated the insulin signaling pathway, promoted the metabolism of protein, fat, and carbohydrate, and improved nutrition metabolism. Our study provides new support for the understanding of the beneficial effect of TiO_2_ NPs on silkworm nutrient metabolism.

## Abstract (precis)

**Summary:** Our study indicates that TiO_2_ nanoparticles promote nutrient metabolism in the fat body of silkworms, and provide a reference for studies investigating the biological function of TiO_2_ nanoparticles.

## INTRODUCTION

The silkworm, which belongs to Lepidoptera: Bombycidae, has complete metamorphosis including four stages: egg, larva, pupa and adult. The larvae use absorbed nutrients to provide energy for the growth, development and metamorphosis, during which they synthesize and secrete a large amount of silk proteins (Tanaka et al., 2009; Truman and Riddiford, 1999). The fat body is the silkworm's intermediate metabolic organ, playing important physiological roles in nutrient storage and transport (Arrese and Soulages, 2010), metabolic detoxification (Cheng et al., 2006a,b; Wen et al., 2003; Petersen et al., 2001), and immune regulation (Trenczek and Faye, 1988).

TiO_2_ nanoparticles (NPs) are a nanomaterial widely used as an environmental cleanup material (Choi et al., 2006; Esterkin et al., 2005), in daily necessities (Weir et al., 2012), and in chemicals (Li et al., 2015a,b; Zhu et al., 2007). It has been shown that feeding silkworms with TiO_2_ NPs can increase feed efficiency and promote silk protein synthesis (Ni et al., 2015). TiO_2_ NPs can mediate the synthesis of ecdysterone to affect silkworm growth and development (Li et al., 2014), and enhance silkworm's anti-viral ability (Su et al., 2014).

The insulin signaling pathway (see http://www.genome.jp/kegg-bin/show_pathway?map04910 for details) participates in the regulation of insect growth and metabolism (Jia et al., 2004). It regulates silkworm glucose metabolism at different developmental stages (Wu and Brown, 2006; Iwami, 2000; Satake et al., 1997), mediates lipid metabolism (Saltiel and Kahn, 2001), and promotes protein synthesis (Douglas et al., 1991). Whether feeding silkworms with TiO_2_ NPs affects the insulin signaling pathway in the fat body is as yet unknown. This study profiled the gene expression in silkworm fat body after TiO_2_ NPs treatment and analyzed regulations of the differentially expressed genes to explore the mechanism of TiO_2_ NPs' effect on silkworm nutrient metabolism.

## RESULTS

### Effect of TiO_2_ NPs on the gene expression in silkworm fat body

**Fig. 1.**
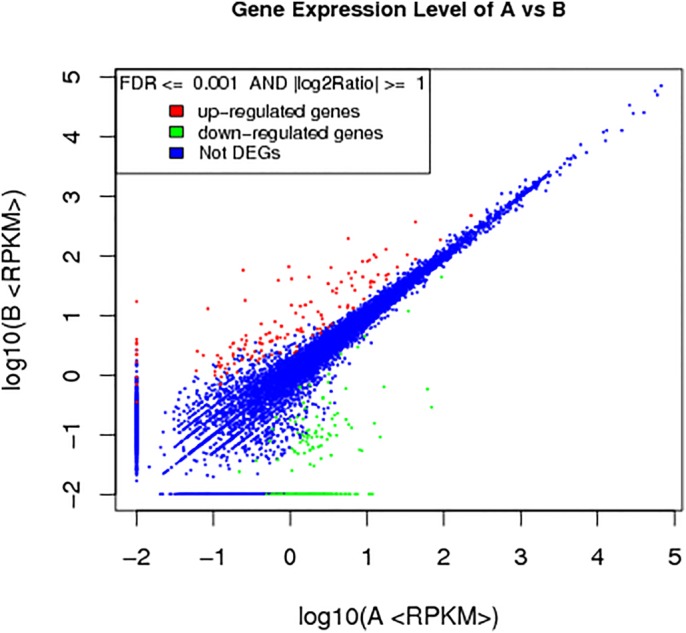
**Statistical chart of significantly differentially expressed genes.** A represents the control group, while B represents the experimental group. RPKM indicates the gene expression in samples. FDR (false discovery rate) is a method to determine the threshold of *P*-values in multiple tests: assume that we have picked R differentially expressed genes, in which S genes really show differential expression and the other V genes are false positive. We use ‘FDR ≤0.001 and the absolute value of log2Ratio ≥1’ as the threshold to determine the significance of differences in gene expression. Red represents the upregulated genes in the figure; green represents downregulated genes; blue represents genes without significant differences.

Fifth instar silkworms were fed with TiO_2_ NPs, and RNA-seq was used to determine TiO_2_ NPs' effect on gene expression in the silkworm fat body. RNA-seq revealed that TiO_2_ NPs treatment led to differential expression of 11,268 genes in the silkworm fat body. 341 genes showed significant differences, among which 138 were upregulated and 203 were downregulated (Fig. 1).

**Fig. 2.**
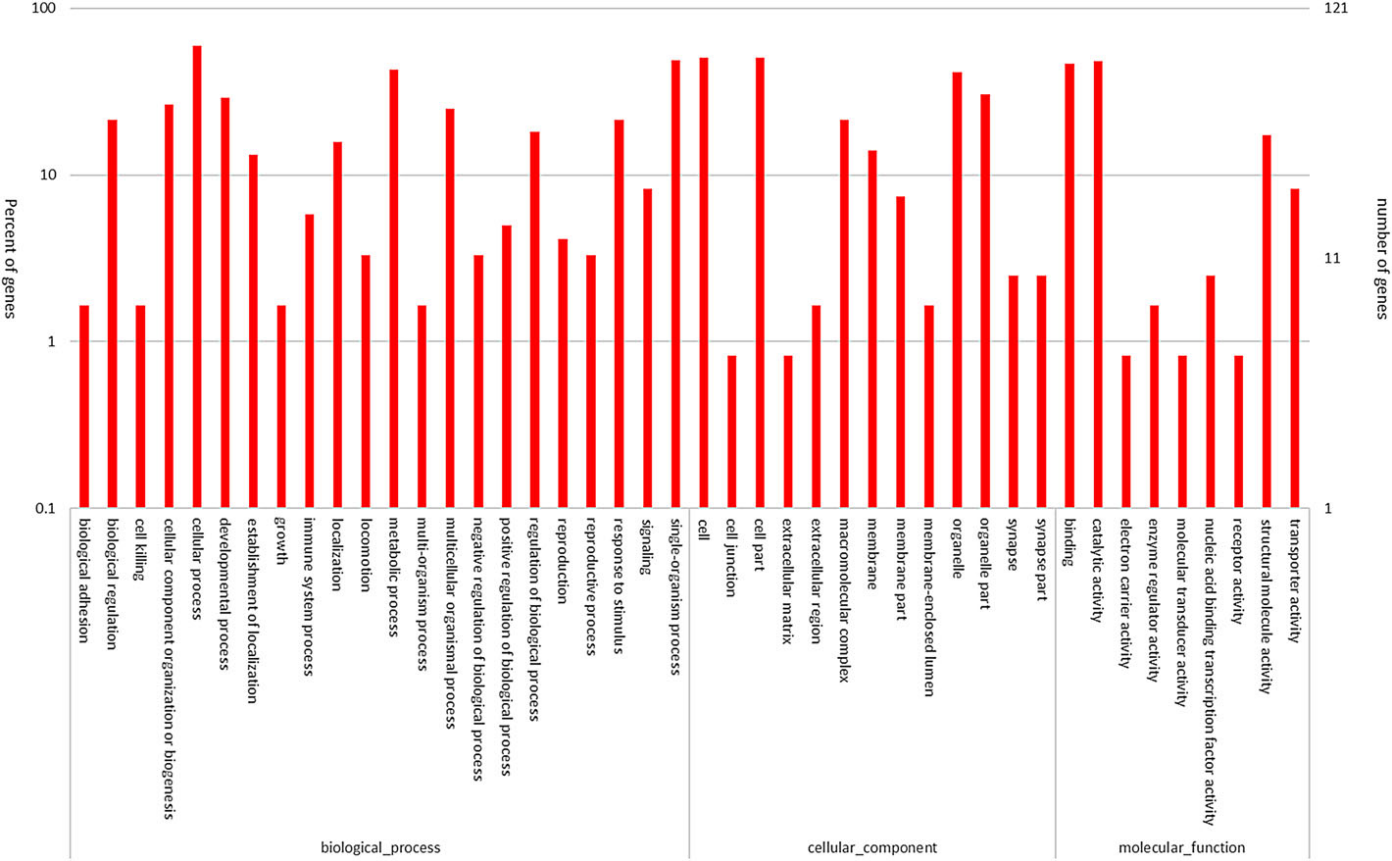
**Functional classification of significantly differentially expressed genes.** A represents the control group while B represents the TiO_2_ NP treatment group. The right ordinate represents the number of genes, with the maximum value of 121 indicating that a total of 121 genes underwent GO function classification. The left vertical axis represents the percentage of genes, indicating the percentage of functional genes to all annotated genes.

Blast2GO program was used to obtain the gene ontology (GO) annotation of differentially expressed genes (DEGs). GO function statistical analysis was done to understand the distribution of gene functions at macro level. As shown by the GO functional classification map (Fig. 2), differentially expressed genes were classified by biological processes, cellular component and molecular functions. Eleven biological processes accounted for more than 10% of the annotated genes, with cellular process, metabolic process and single-organism process showing the highest percentages of annotated genes. Five cellular component subgroups accounted for more than 10% of annotated genes, with cell, cell part and organelle showing the highest percentages of annotated genes. Three molecular functions accounted for 10% or more of annotated genes, which were binding, catalytic activity and structural molecular activity.

**Fig. 3.**
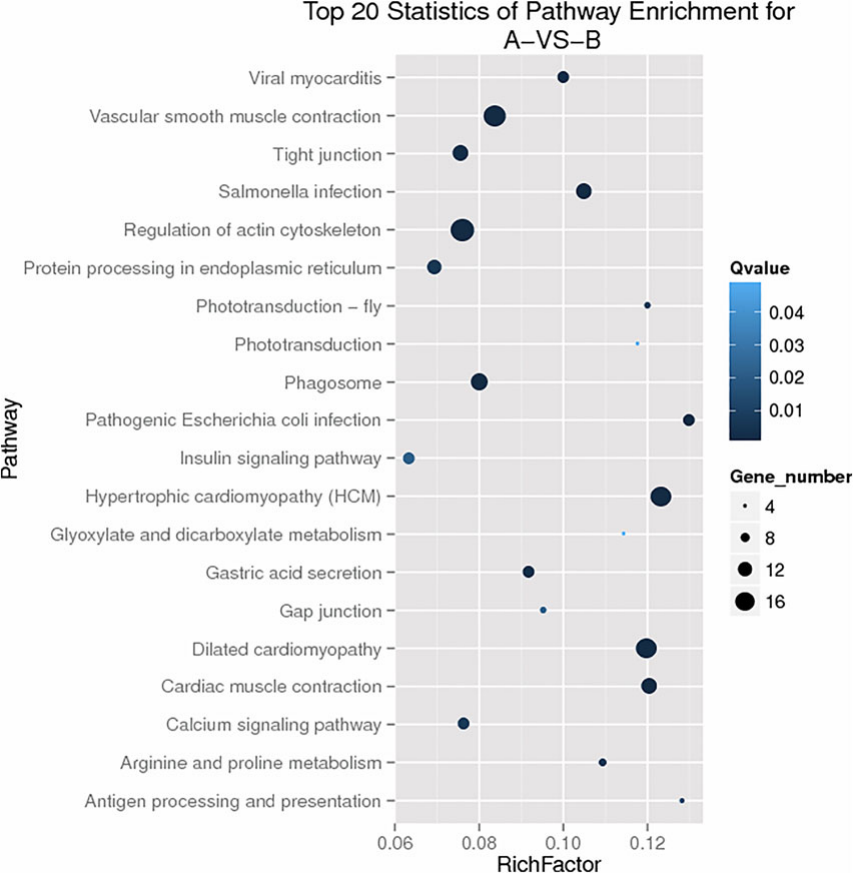
**Scatter plot of KEGG pathway enrichment statistics.** A represents control group, and B represents experimental group. Rich factor is the ratio of numbers of differentially expressed genes annotated in this pathway term to the numbers of all genes annotated in this pathway term. Greater rich factor means greater intensiveness. Q-value is corrected *P*-value ranging from 0∼1, with a lower value means greater intensiveness. Top 20 pathway terms enriched are displayed in the figure.

The results of KEGG enrichment analysis were graphically displayed to analyze the enrichment patterns of differentially expressed genes in different pathways. KEGG enrichment scatterplots (Fig. 3) indicated significant enrichments of all differentially expressed genes (Q-value <0.05). Multiple pathways found have not been previously studied. However, the insulin signaling pathway has been shown to mediate insect nutrient metabolism and growth/development, participate in maintaining the homeostasis of lipids and carbohydrates, and affect protein synthesis. Therefore, investigating the transcriptional expression profiles of insulin signaling pathway genes carries important significance for the elucidation of TiO_2_ NPs' positive effect on silkworm nutrient metabolism.

### RNA-seq results and qRT-PCR validation of important differentially expressed genes

Based on RNA-seq results, RPKM gene expression levels were analyzed along with KEGG pathway information to determine expression level changes of insulin signaling pathway genes (troponin C, troponin C 25D), protein metabolism related genes (4-hydroxyphenylpyruvae dioxygenase, creatine kinase, threonine dehydratase), lipid metabolism related genes (fatty acid synthase, P270, fatty acid desaturase, fatty acid transport protein), carbohydrate metabolism related genes (facilitated trehalose transporter Tret1-2 homolog, facilitated trehalose transporter Tret1), and heat shock protein genes (*hsp* 68, *hsp* 1, *hsp* 20.4, *hsp* 20.8, *hsp* 16.1/*hsp* 16.11, *hsp* 90, *hsp* 70). As shown in Table 1 after feeding with TiO_2_ NPs, the expression levels of troponin C and troponin C 25D were 10.98-fold and 2.93-fold, respectively, higher than those of the control group. The expression levels of protein synthesis related genes 4-hydroxyphenylpyruvate dioxygenase and creatine kinase were increased by 13.67 times and 3.82 times, respectively, and threonine dehydratase's level was decreased by 103 times. The expression levels of lipid metabolism related genes fatty acid synthase, P270, and fatty acid desaturase were increased by 6.47-fold, 4.62-fold, and 1.18-fold, respectively, and fatty acid transport protein level was decreased by 1.18-fold. The expression levels of carbohydrate metabolism related genes Tret1 and Tret1-2 homolog were increased by 9.31-fold and 4.57-fold. The expression levels of heat shock protein genes *hsp* 68, *hsp* 1, *hsp* 20.4, *hsp* 20.8, *hsp* 16.1/ *hsp* 16.11, *hsp* 90, and *hsp* 70 were 17.10-fold, 6.76-fold, 3.09-fold, 2.63-fold, 2.61-fold, 2.10-fold and 2.03-fold higher, respectively, than those of the control group. These results indicated that TiO_2_ NP treatment can significantly increase the expression of insulin signaling pathway and nutrient metabolism related genes and that of heat shock proteins.

**Table 1.**
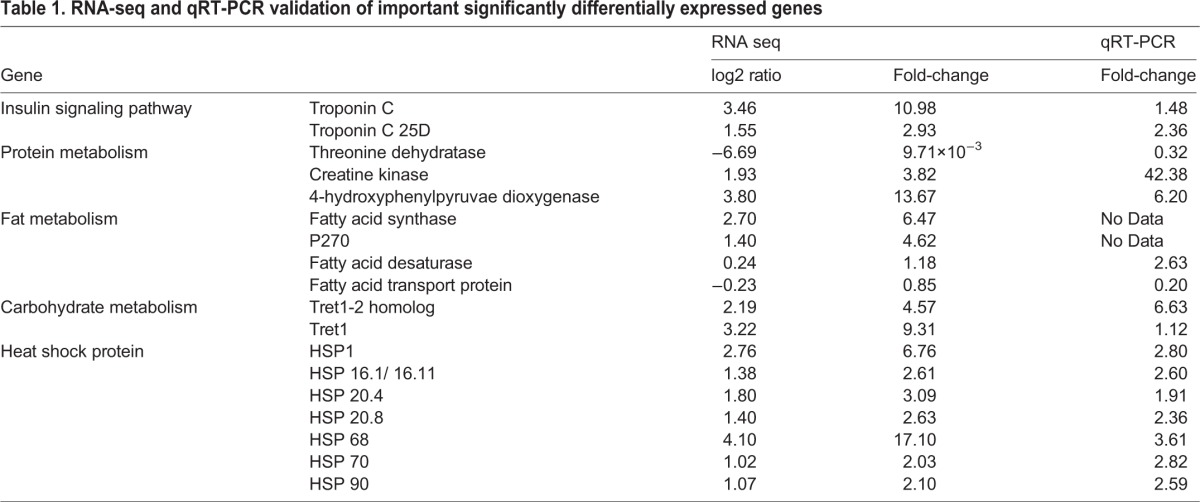
**RNA-seq and qRT-PCR validation of important significantly differentially expressed genes**

The accuracy of the RNA-seq results was validated by doing qRT-PCR for the above genes, which showed consistent results (Table 1). The insulin signaling pathway genes troponin C 25D and troponin C were upregulated by 2.36-fold and 1.48-fold, respectively. Protein synthesis related genes creatine kinase and 4-hydroxyphenylpyruvate dioxygenase were upregulated by 42.38-fold and 6.20-fold, respectively, while threonine dehydratase's expression was downregulated by 3.09-fold. The lipid metabolism-related genes fatty acid desaturase and fatty acid transport protein were upregulated by 2.63-fold and downregulated by 5.12-fold, respectively. Carbohydrate metabolism related genes Tret1-2 homolog and Tret1 were upregulated by 6.63-fold and 1.12-fold, respectively. The expression levels of heat shock protein genes *hsp* 68, *hsp* 70, *hsp* 1, *hsp* 16.1/*hsp* 16.11, *hsp* 90, *hsp* 20.8 and *hsp* 20.4 were upregulated by 3.61-fold, 2.82-fold, 2.80-fold, 2.60-fold, 2.59-fold, 2.36-fold, and 1.91-fold, respectively. No effective data were obtained for fatty acid synthase gene and P270 gene due to their extremely low expression. These results indicate the high accuracy of RNA-seq data.

### Measurements of contents of trehalose, triglyceride, total proteins, and lipase activity in silkworm fat body

To investigate whether TiO_2_ NPs affects the nutrient metabolism of silkworm fat body, we determined fat body trehalose content, triglyceride content, lipase activity, and total protein content. As shown in Table 2, three days after TiO_2_ NP feeding, triglyceride content and lipase activity were increased by 0.94-fold and 3.86-fold, respectively, compared with that of the control groups, indicating that TiO_2_ NPs can improve adipolysis metabolism by increasing lipase activity and promoting fat hydrolysis. The content of trehalose of the experimental group was increased by 1.33-fold compared with that of the control group, indicating TiO_2_ NPs feeding can improve silkworm carbohydrate metabolism by promoting trehalose synthesis. The experimental group's total protein amount was 1.21-fold higher than that of the control group, indicating that TiO_2_ NPs can improve silkworm protein metabolism by promoting protein synthesis.

**Table 2.**
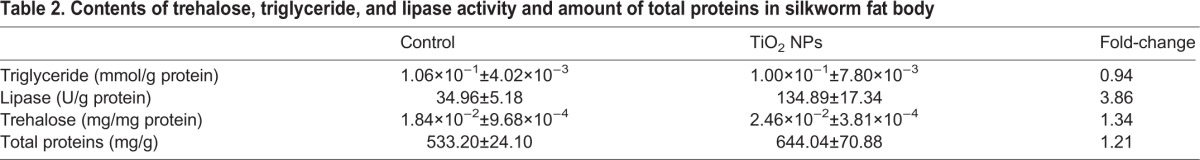
**Contents of trehalose, triglyceride, and lipase activity and amount of total proteins in silkworm fat body**

## DISCUSSION

### Profiles of gene expression in silkworm fat body after TiO_2_ NP treatment

Previous studies have shown that continuous feeding of low-dose TiO_2_ NPs can enhance silkworm resistance (Wang et al., 2015), promote silkworm growth and development (Li et al., 2015a,b), and increase fibroin synthesis (Ni et al., 2015). As the silkworm's central metabolism tissue, the fat body is where nutrient synthesis, conversion, utilization, and storage occur (Arrese and Soulages, 2010) and participates in insect growth/development and longevity (Rusten et al., 2004; Hwangbo et al., 2004). RNA-seq has been used for the determination of gene expression (Levin et al., 2010), discovery and identification of unknown genes (Roberts et al., 2011), detection of fusion genes (Maher et al., 2009), identification of single nucleotide variations (Barbazuk et al., 2007), and co-expression network analysis (Giorgi et al., 2013). In this study, RNA-seq technology was used for the first time to explore the expression profiles of genes in silkworm fat body after TiO_2_ NP treatment. The established database provides reference for the research of other insects' fat body genes.

### Regulation of nutrient metabolism and insulin signaling pathway

The insulin signaling pathway participates in lipid and carbohydrate homeostasis, directly affects the synthesis of proteins, lipids, and carbohydrates, and regulates cell proliferation and apoptosis. For the upregulated target genes, troponin C 25D and troponin C can promote glycogen synthesis to mediate carbohydrate metabolism, 4-hydroxyphenylpyruvate dioxygenase can enhance amino acid metabolism (Knox and LeMay-Knox, 1951), creatine kinase promotes the synthesis of glycine and improve energy transfer efficiency (Wallimann et al., 1992; Wallimann and Hemmer, 1994), fatty acid synthase and P270 promotes fatty acid synthesis (Ueno, 2000), fatty acid desaturase enhances fat metabolism, Tret1 and Tret1-2 can promote the transportation of trehalose. For the downregulated target genes, threonine dehydratase can decrease the degradation of threonine and serine, fatty acid transport protein can promote the synthesis and storage of other nutrients from fatty acids. Triglyceride is an important storage molecule of energy and water in insects, which can be used as energy and fat transporter (Drummond and Brefere, 2001). Fatty acid synthase's upregulation promotes the synthesis of fatty acids, the major components for fat synthesis; lipase can hydrolyze triglyceride, thus lipase and triglyceride content indicates fat metabolic level (Haemmerle et al., 2006). In this study, we found that the lipase activity of the experimental group was 3.86-fold higher than that of the control group, and the experimental group's triglyceride content was 0.94-fold that of the control group, which indicates that TiO_2_ NPs can enhance lipid metabolism and promote the conversion of lipids to other nutrients. This study, for the first time, explored the regulatory effect of TiO_2_ NPs on silkworm insulin signaling pathway and its upregulation of nutrient metabolism. It provides a new direction for the research on fat body of silkworm and other insects.

### Expression characteristics of HSP genes

Heat shock proteins (HSPs) have functions of maintaining protein activities, ensuring correct protein folding as chaperones, and participating in immune response during heat shock. In this study, we found that the upregulation of *hsp* 68, *hsp* 70, and *hsp* 90 was positively correlated with enhanced protein metabolism. Under high temperature, hypertonic, toxic and starvation conditions, silkworm's survival is directly related with trehalose (Strom and Kaasen, 1993). This study showed that TiO_2_ NP treatment led to increased trehalose content in silkworm fat body, along with upregulated HSPs, indicating a synergy between trehalose and HSPs in stress response. However, the mechanism of this synergy needs to be clarified in future studies.

It has been shown that HSP70 has anti-apoptotic effects (Mosser et al., 1997), and HSP70 and HSP90 can regulate the JNK pathway in cell proliferation and apoptosis (Gabai et al., 1998; Zhang et al., 2010); HSP90 can regulate AKT expression and co-function with AKT to induce apoptosis. The RNA-seq results in this study indicated that the insulin signaling pathway can regulate autophagy and affect nutrition metabolism. Heat shock proteins are involved in prostate cancer pathway and MAPK pathway. The relationship between insulin signaling pathway and MAPK pathway and the relationship between AKT and apoptosis suggest the important interaction between insulin signaling pathway and HSPs in the growth and viability of silkworms. The underlining mechanisms of the regulations and interactions require further studies.

### Conclusion

TiO_2_ NP treatment affects the gene expression of the insulin signaling pathway and improves silkworm nutrient metabolic levels.

## MATERIALS AND METHODS

### Insects strains

The silkworm variety used was Jingsong×Haoyue, preserved in our laboratory. The feeding conditions were 25°C, 12 h light/12 h darkness, and feeding with mulberry leaves three times daily. All the experimental animals comply with all relevant institutional and national animal welfare laws, guidelines and policies.

### Chemicals

Anatase TiO_2_ NPs was purchased from Hanzhou Wanjing Ltd. and prepared as 5 g/l stock solution for use when diluted to 5 mg/l (Zhang et al., 2014).

### Treatments

Fifth instar silkworms were divided into two groups, and each group had three replicates of 30 silkworms. The leaves for the control group were sprayed with water, and those of the experimental group were sprayed with 5 mg/l TiO_2_ NPs solution; all leaves were air-dried before feeding for 3 days.

### Sample preparation and RNA-seq analysis

The fat bodies of 20 randomly selected silkworms in each group replicate were collected and saved at −80°C for further analyses.

Total RNA samples were first treated with DNase I to eliminate possible DNA contamination. The mRNAs were enriched by using oligo(dT) magnetic beads (for eukaryotes). After mixed with the fragmentation buffer, the mRNAs were fragmented into short fragments (about 200 bp). Then the first strand of cDNA was synthesized by using random hexamer-primer. Reaction buffer, dNTPs, RNase H and DNA polymerase I were added to synthesize the second strand. Double strand cDNAs were purified with magnetic beads. End reparation and 3′-end single nucleotide A (adenine) addition were then performed. Finally, sequencing adaptors were ligated into the fragments. The fragments were enriched by PCR amplification. During the QC step, Agilent 2100 Bioanaylzer and ABI StepOnePlus Quantitative RT-PCR System were used to qualify and quantify the sample library. The library products were sequenced via Illumina HiSeq™ 2000 or other sequencer when necessary by Beijing Genomics Institute (BGI) (Shenzhen, China).

### Quantitative RT-PCR analysis

Primer 6.0 (http://www.premierbiosoft.com/primerdesign/index.html) was used for the design of qRT-PCR primers for the important differentially expressed genes (Table 3) with actin 3 as the reference gene. qRT-PCR was performed using the Viia 7 Quantitative RT-PCR System (ABI) with SYBR Premix Ex Taq™ (Takara) following the manufacturer's instructions. The reaction conditions were denaturation at 95°C for 1 min and 45 cycles of 95°C for 5 s, 55°C for 10 s, and 72°C for 10 s. All samples were measured independently three times. The qPCR data were analyzed by using Microsoft Excel 2010 (Microsoft Corporation, Redmond, WA, USA).

**Table 3.**
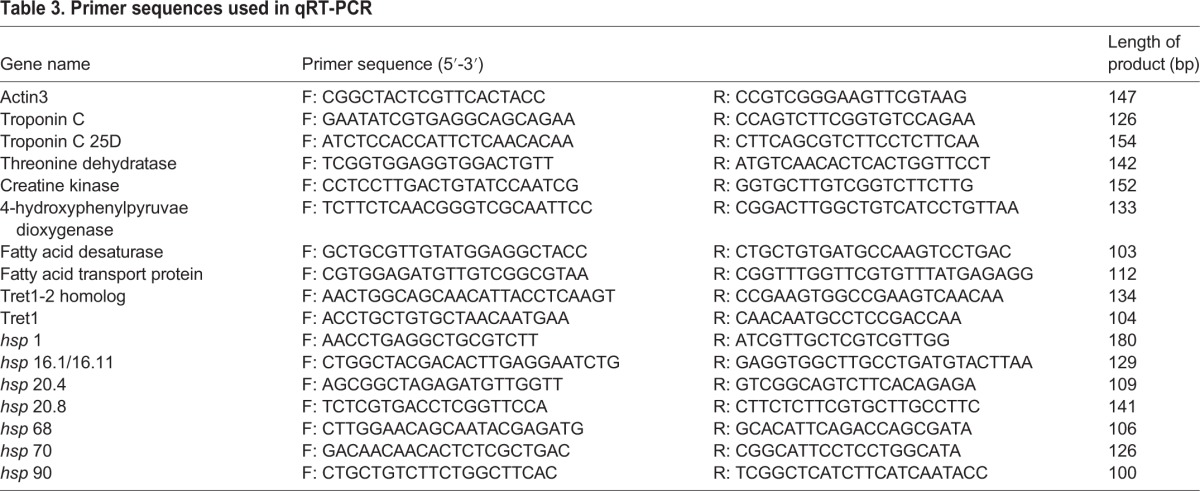
**Primer sequences used in qRT-PCR**

### Measurements of trehalose, triglyceride contents, and lipase activity and total protein content

Trehalose content was determined using a trehalose kit purchased from Suzhou Keming Biotechnology Co. following the manufacturer's manual: 0.1 g fat body tissue was weighed and ground and mixed with 1 ml extraction solution; after standing for 45 min, the mixture was centrifuged at 6300 ***g*** for 10 min; the supernatant was mixed with working solution at a ratio of 1:4 before being boiled at 100°C for 10 min; OD_620_ was measured after the mixture was cooled down to room temperature.

Triglyceride content was determined using triglyceride (TG) kit purchased from Nanjing Jiancheng Biotechnology Institute following the manufacturer's manual: 0.1 g fat body tissue was mixed with 900 μl ethanol and homogenized on ice; the mixture was centrifuged at 610 ***g*** for 10 min; the supernatant was mixed with working solution at a ratio of 1:100 and incubated at 37°C for 10 min, before OD_510_ was measured.

Lipase activity was determined using lipase (LPS) kit purchased from Nanjing Jiancheng Biotechnology Institute following the manufacturer's manual: 0.1 g fat body tissue was mixed with 400 μl ethanol and homogenized on ice; the mixture was centrifuged at 610 ***g*** for 10 min; the substrate buffer was pre-heated to 26°C for 5 min, and 2.5 μl supernatant was mixed with 2.5 μl solution IV and 200 μl substrate buffer to determine OD_420_ A1; OD_420_ A2 was measured after 10 min of incubation at 26°C for the calculation of ΔA.

The content of total proteins was determined using BCA kit purchased from Shanghai Biotechnology Ltd following the manufacturer's manual: 0.03 g fat body tissue was mixed with 1 ml PBS and homogenized on ice; the mixture was centrifuged at 6300 ***g*** for 10 min; the supernatant was mixed with PBS and working solution and incubated at 37°C for 30 min, before OD_562_ was measured.
